# Imaging Cardiac Amyloidosis: From Early Diagnosis to Risk Stratification and Evaluation of Treatment Efficacy

**DOI:** 10.3390/jcdd13080401

**Published:** 2026-08-21

**Authors:** Matteo Sclafani, Domitilla Russo, Georgios Oikonomou, Giovanni Camastra, Emanuela Belmonte, Giacomo Tini, Rossella Rotunno, Cristina Chimenti, Chiara Lanzillo, Beatrice Musumeci, Teresa Castiello, Stefano Regondi, Roberto Ricci, Luca Cacciotti, Luca Arcari

**Affiliations:** 1 Royal Brompton Hospital, Guy’s and St Thomas’ NHS Foundation Trust, London SW3 6PY, UK; matteo.sclafani18@gmail.com (M.S.); georgios.oikonomou1@nhs.net (G.O.); 2 Cardiology Unit, Department of Clinical and Molecular Medicine, Sant’Andrea University Hospital, Sapienza University, 00189 Rome, Italy; giacomo.tinimelato@uniroma1.it (G.T.); beatrice.musumeci@uniroma1.it (B.M.); 3 UOC Cardiology, Santo Spirito Hospital, 00193 Rome, Italy; russodomitilla@gmail.com (D.R.); chiara.lanzillo@aslroma1.it (C.L.); roberto.ricci@aslroma1.it (R.R.); 4 Hellenic Heart Foundation (ELIKAR), 115 21 Athens, Greece; 5 Cardiology Unit, Madre Giuseppina Vannini Hospital, 00177 Rome, Italy; gcamastra@virgilio.it (G.C.); ebelmonte.1@gmail.com (E.B.); rossella.rotunno@libero.it (R.R.); stefano.regondi@figliesancamillo.it (S.R.); lcuccc@libero.it (L.C.); 6 Department of Medical and Cardiovascular Sciences, Sapienza University of Rome, 00185 Rome, Italy; cristina.chimenti@uniroma1.it; 7 MIAL Healthcare, London E9 6SR, UK; teresa.castiello@mial.healthcare; 8 Homerton University Hospital, London W1G 8QL, UK

**Keywords:** cardiac amyloidosis, echocardiography, cardiac magnetic resonance imaging, T1 mapping, cardiac computed tomography, bone scintigraphy

## Abstract

Cardiac amyloidosis (CA) is an infiltrative cardiomyopathy caused by extracellular deposition of misfolded proteins, most commonly immunoglobulin light chains (AL) or transthyretin (ATTR). Once considered a rare disease, CA is increasingly recognised due to improved diagnostic strategies and the availability of disease-modifying therapies. Early diagnosis is crucial, as treatment efficacy and clinical outcomes are strongly influenced by the stage of cardiac involvement. Multimodality cardiac imaging plays a central role in the diagnostic pathway, risk stratification, and evaluation of therapeutic response in CA. Echocardiography represents the first-line imaging modality and is essential for raising clinical suspicion through the identification of characteristic structural and functional abnormalities, including ventricular wall thickening, diastolic dysfunction, and distinctive strain patterns. Bone scintigraphy has revolutionised the non-invasive diagnosis of ATTR-CA, allowing accurate identification of transthyretin-related disease in the absence of monoclonal gammopathy, which needs to be excluded via serum and urinary immunofixation. Cardiovascular magnetic resonance provides advanced tissue characterisation through late gadolinium enhancement and quantitative mapping techniques, enabling detection of early myocardial involvement and robust prognostic stratification. Emerging imaging modalities, including dual-energy (spectral) computed tomography and positron emission tomography tracers, show promise in myocardial amyloid quantification and subtype differentiation, although their role is still evolving. Integration of imaging findings with clinical and laboratory parameters allows comprehensive disease assessment, facilitating early diagnosis, guiding therapeutic decisions, and improving risk stratification. This review summarises the current role of multimodality imaging in CA, highlighting its contribution from early detection to prognostic evaluation and monitoring of treatment efficacy, with particular emphasis on the emerging role of quantitative imaging in monitoring treatment response.

## 1. Introduction

Amyloidosis is a systemic disorder characterised by extracellular deposition of misfolded proteins that are resistant to physiological degradation, leading to progressive dysfunction of multiple organs, including the heart [1]. Cardiac amyloidosis (CA) results from myocardial infiltration by amyloid fibrils, predominantly derived either from immunoglobulin light chains produced by plasma cell dyscrasia (AL amyloidosis) or from transthyretin (TTR) protein misfolding (ATTR amyloidosis). ATTR amyloidosis may arise from pathogenic variants of the TTR gene (variant ATTR (ATTRv)) or from age-related instability of the wild-type protein (wild-type ATTR (ATTRwt)). Histologically, amyloid deposition appears as extracellular, amorphous, eosinophilic material accumulating within the myocardial interstitium and vessel walls [2]. These deposits exhibit characteristic apple-green birefringence when stained with Congo red under polarised light. Progressive amyloid accumulation leads to cardiomyocyte atrophy, interstitial expansion, and increased myocardial stiffness, ultimately resulting in restrictive cardiomyopathy and heart failure [3]. Despite increasing recognition, CA remains substantially underdiagnosed. Screening studies have demonstrated a higher-than-expected prevalence of CA in specific populations, including elderly patients with aortic stenosis and individuals with bilateral carpal tunnel syndrome [4,5]. The recent development of disease-modifying therapies, particularly for ATTR-CA, has significantly changed the clinical landscape, emphasising the importance of early identification and timely treatment initiation, which are associated with improved clinical outcomes [6,7]. Cardiac imaging plays a central role throughout the diagnostic and management pathway of CA, from raising initial suspicion to quantifying myocardial infiltration and monitoring the response to therapy. The aim of the present review is to summarise the role of multimodality cardiac imaging in CA, with a particular focus on its contribution to early diagnosis, risk stratification, and evaluation of treatment response. Although several reviews have addressed cardiac imaging in CA, the present work is deliberately structured around three clinically actionable questions (the contribution of imaging to early diagnosis, risk stratification, and monitoring of treatment response) and maps individual imaging parameters to each of these domains rather than providing a purely descriptive catalogue of imaging findings. Particular emphasis is placed on quantitative and emerging techniques whose role in longitudinal disease monitoring is only now being defined.

## 2. Echocardiography

Echocardiography is typically the first-line imaging modality in the evaluation of suspected CA because of its widespread availability, low cost, and ability to identify characteristic morphological and functional abnormalities. The most common echocardiographic features, summarised in Table 1, reflect myocardial amyloid infiltration and include increased left ventricular (LV) and right ventricular wall thickness, increased LV mass, and thickening of the atrioventricular valves and interatrial septum [8].

Because LV wall thickening results from extracellular amyloid deposition rather than true myocyte hypertrophy, a discordance between increased wall thickness on echocardiography and low QRS voltages on electrocardiography is frequently observed and represents a classic diagnostic red flag. Additional typical findings include advanced diastolic dysfunction due to impaired ventricular relaxation and reduced compliance, often disproportionately severe relative to the degree of LV wall thickening, small LV cavity size, mild pericardial effusion, and bi-atrial enlargement.

Atrial dilatation reflects not only chronically elevated LV filling pressures but also direct atrial amyloid infiltration, leading to atrial mechanical dysfunction [9]. This atrial myopathy promotes blood stasis and confers a high risk of intracardiac thrombus formation, even in patients in sinus rhythm [10,11], thereby increasing the risk of systemic embolism despite appropriate anticoagulation [12]. In this context, speckle-tracking echocardiography provides incremental value for assessment of left atrial (LA) function. LA phasic strain parameters, including reservoir, conduit, and booster pump strain, as well as peak longitudinal strain, are markedly impaired in CA and reflect atrial mechanical dysfunction [13].

**Figure 1:**
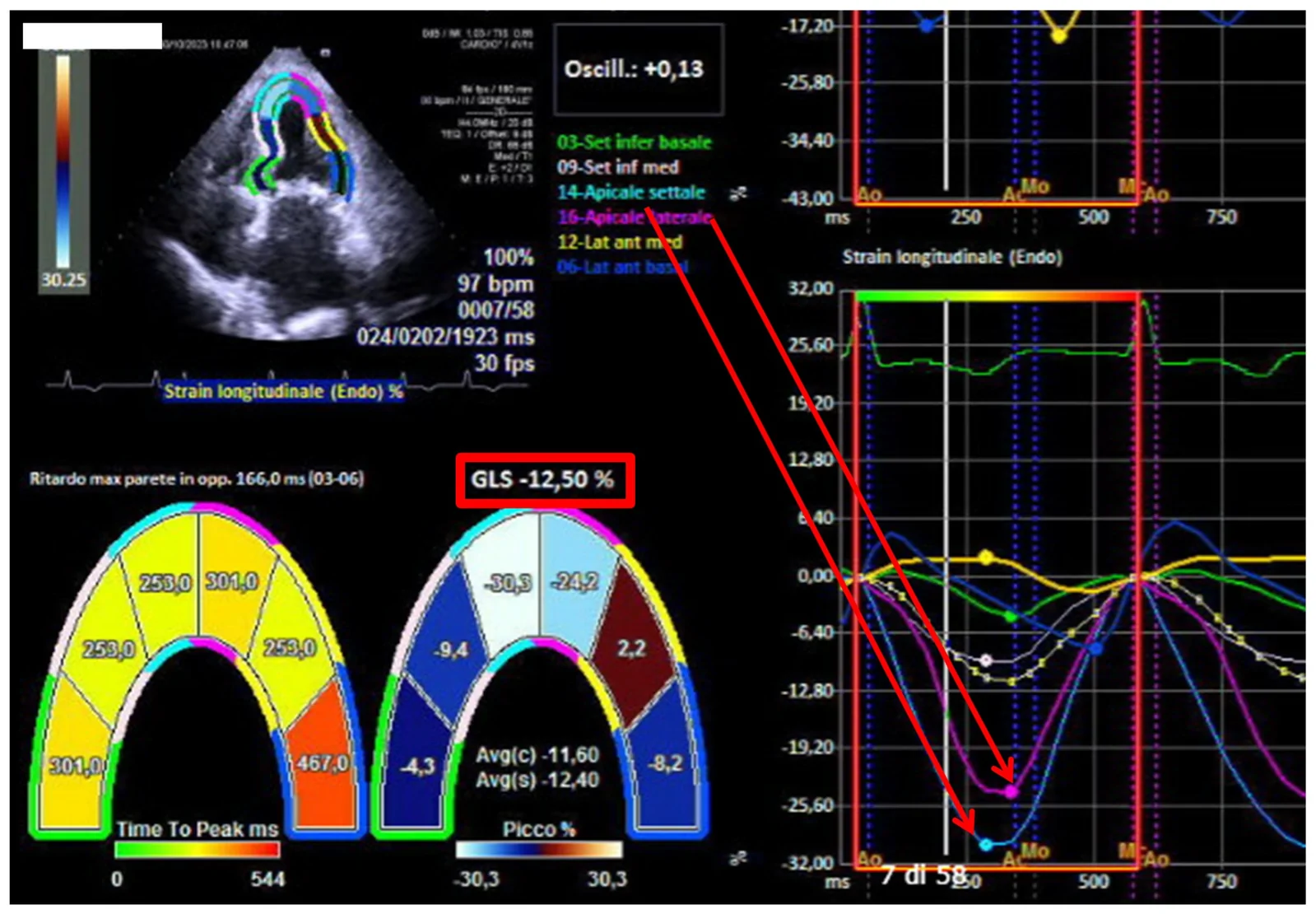
Speckle-tracking echocardiography; 4-chamber view. Impaired global longitudinal strain (−12.5%) with preserved deformation of septal and lateral apical segments (arrow), depicting the typical “apical sparing” pattern in a patient with TTR cardiac amyloidosis.

Left ventricular strain analysis also plays a pivotal role in the echocardiographic assessment of CA. Although global impairment of longitudinal, radial, and circumferential strain may be observed, these findings are not disease-specific. In contrast, the characteristic base-to-apex gradient of longitudinal strain, with a severe reduction in basal segments and relative preservation of apical function, the so-called “apical sparing” or “cherry-on-top” pattern, is highly sensitive and specific to CA [14] (Figure 1; Video S1). Importantly, a reduction in global longitudinal strain may be detected before a decline in LV ejection fraction, which is often preserved in the early stages of the disease [15]. As CA progresses, systolic function deteriorates, with reductions in ejection fraction, stroke volume, and myocardial contraction fraction [8].

Echocardiography may also identify associated conditions that should raise suspicion for CA. In particular, the presence of paradoxical low-flow, low-gradient aortic stenosis with preserved LV ejection fraction, especially when accompanied by other echocardiographic red flags, is strongly associated with transthyretin amyloidosis and warrants further investigation [16].

Historically, a granular or “sparkling” myocardial appearance was considered a specific feature of CA. However, this finding has limited diagnostic value in contemporary practice, as harmonic imaging and gain settings may produce similar appearances in the absence of amyloid infiltration. Consequently, myocardial sparkling is now regarded as a nonspecific and unreliable marker [8].

Beyond its diagnostic role, echocardiography provides prognostic information in CA. Reduced tricuspid annular plane systolic excursion (TAPSE), impaired right ventricular strain, and reduced LA longitudinal strain have been consistently associated with increased mortality in both AL and ATTR amyloidosis [17,18]. In AL amyloidosis, additional parameters associated with worse prognosis include increased LA size, reduced stroke volume, and impaired global longitudinal strain [17,19,20].

Overall, echocardiographic abnormalities in CA reflect the extent of amyloid burden affecting both ventricular and atrial myocardium and provide essential diagnostic and prognostic information within a multimodality imaging framework.

## 3. Bone Scintigraphy

Bone scintigraphy plays a pivotal role in the non-invasive diagnosis of ATTR-CA. The technique employs technetium-99m-labelled bone-seeking radiotracers, including ^99m^Tc–3,3-diphosphono-1,2-propanodicarboxylic acid (DPD), ^99m^Tc-pyrophosphate (PYP), and ^99m^Tc-hydroxymethylene diphosphonate (HMDP). Under physiological conditions, approximately 50% of these tracers localise to the skeleton, with the remainder rapidly cleared from soft tissues through renal excretion [21]. In the presence of CA, however, abnormal myocardial tracer uptake is observed.

The mechanism underlying myocardial uptake is thought to involve binding of bone tracers to microcalcifications associated with amyloid fibrils, which are more abundant in transthyretin-derived deposits than in light-chain amyloid. Accordingly, ATTR-CA demonstrates high avidity for bone tracers, whereas AL amyloidosis typically shows absent or low-grade myocardial uptake [22,23].

Myocardial tracer uptake is visually graded using the Perugini scale, ranging from grade 0 (no uptake) to grade 3 (myocardial uptake greater than bone uptake), based on comparison between myocardial and rib activity [23]. Proper acquisition, interpretation, and integration of bone scintigraphy within the diagnostic algorithm are essential to ensure accurate diagnosis of ATTR-CA.

Because bone tracers also exhibit blood pool activity, planar imaging alone is insufficient for reliable interpretation. Single-photon emission computed tomography (SPECT) is mandatory to differentiate true myocardial uptake from residual blood pool activity or extracardiac uptake, thereby reducing the risk of false-positive results [23]. Additional causes of false-positive myocardial uptake include acute or subacute myocardial infarction, which typically results in focal tracer accumulation, and rare forms of amyloidosis such as apolipoprotein A1-related disease.

A non-invasive biopsy-free diagnosis of ATTR-CA can be established only when grade 2 or 3 myocardial uptake on bone scintigraphy is observed in patients with echocardiographic and/or CMR findings suggestive of CA, in the absence of a detectable monoclonal gammopathy on serum and urine testing (Gillmore diagnostic algorithm). Failure to adequately exclude AL amyloidosis represents the most common cause of misdiagnosis.

False-negative scans may occur in early disease stages, when myocardial amyloid burden is low, in patients carrying specific TTR mutations such as Phe64Leu or Val30Met [24], or when image acquisition and processing are suboptimal [2]. Despite these limitations, bone scintigraphy demonstrates excellent sensitivity for the identification of ATTR-CA.

Bone scintigraphy currently has no established prognostic role, as myocardial tracer uptake graded by the Perugini score has not been shown to correlate with clinical outcomes [25]. Nevertheless, emerging evidence suggests that diffuse right ventricular uptake of bone-seeking tracers on SPECT imaging may be associated with adverse outcomes in patients with ATTR-CA [26].

## 4. Magnetic Resonance Imaging

Cardiovascular magnetic resonance (CMR) provides highly accurate and comprehensive characterisation of myocardial morphology, function, and tissue composition, representing a cornerstone imaging modality in CA, from early diagnosis to risk stratification and evaluation of treatment response [27].

CMR allows precise evaluation of cardiac structure and function, typically revealing features consistent with restrictive cardiomyopathy. These include concentric LV wall thickening, frequently more pronounced in basal segments, increased indexed LV mass, and reduced LV cavity size with preserved or mildly reduced LV ejection fraction, often accompanied by decreased stroke volume. Additional findings commonly include bi-atrial enlargement, interatrial septal thickening, valvular thickening, and pericardial effusions [28,29,30].

Although both AL and ATTR forms share several morphological features, subtle differences have been described. Asymmetric LV hypertrophy appears more frequently in ATTR-CA, likely reflecting differential myocardial remodelling patterns between amyloid subtypes [31].

Beyond conventional functional parameters, CMR myocardial strain analysis (derived from feature-tracking or tagging techniques) can detect subtle myocardial dysfunction. Reduced longitudinal and circumferential strain correlates with amyloid burden and may identify early cardiac involvement, potentially preceding late gadolinium enhancement (LGE) abnormalities, particularly in ATTR-CA [32,33].

The major strength of CMR in CA lies in advanced myocardial tissue characterisation, particularly through parametric mapping techniques that allow quantitative assessment of myocardial relaxation properties.

Native T1 mapping quantifies longitudinal relaxation time without contrast administration and reflects combined alterations in intracellular and extracellular compartments. In CA, native T1 values are markedly elevated compared with normal myocardium and other hypertrophic conditions such as hypertrophic cardiomyopathy [28]. Elevated native T1 correlates with disease severity, ventricular dysfunction, and amyloid burden and has demonstrated both diagnostic and prognostic value in AL and ATTR amyloidosis [34,35]. Importantly, native T1 may be particularly useful when gadolinium administration is contraindicated, such as in advanced renal dysfunction, and holds promise as a potential surrogate endpoint in clinical trials.

Extracellular volume (ECV) mapping provides a direct estimate of myocardial extracellular expansion by combining pre- and post-contrast T1 measurements corrected for haematocrit. Because amyloid fibril deposition primarily expands the interstitial space, ECV represents a robust quantitative marker of myocardial amyloid burden [36].

ECV strongly correlates with myocardial tracer uptake on bone scintigraphy and demonstrates high diagnostic accuracy in differentiating CA from other hypertrophic phenotypes [35,37]. Although elevated in both AL and ATTR forms, ECV values are generally higher in ATTR-CA [31,34]. Current consensus statements suggest that ECV values exceeding approximately 40% are highly suggestive of CA [38].

Beyond diagnostic applications, ECV is strongly associated with disease severity and clinical outcomes in both amyloid subtypes and provides incremental prognostic value beyond conventional imaging and clinical parameters [35,39]. Moreover, longitudinal reductions in ECV have been shown to reflect regression of myocardial amyloid deposition following disease-modifying therapies, highlighting its emerging role in monitoring treatment response [40,41]. Proposed risk thresholds range around 44% for AL and 40% for ATTR amyloidosis [42,43]. The clinical implications of ECV quantification have been further extended by recent evidence. In a cohort of 560 patients with newly diagnosed systemic AL amyloidosis undergoing CMR with ECV mapping before chemotherapy, ECV mapping was the only independent predictor of prognosis among the different approaches used to define cardiac involvement, outperforming serum biomarkers and echocardiographic criteria [44]. ECV also refined the interpretation of haematological response, clarifying the relationship between the depth and the speed of response and survival, and identifying the patients in whom achieving a rapid and deep haematological response is most critical [44]. These findings support the transition of quantitative CMR biomarkers from descriptive imaging findings towards clinically actionable tools, capable of redefining cardiac involvement and informing the targets of treatment in AL amyloidosis [44].

Overall, ECV allows characterisation of the continuous spectrum of myocardial infiltration and appears to provide superior diagnostic and prognostic performance compared with LGE alone 37]. Nevertheless, native T1 and ECV should be interpreted according to scanner-specific reference ranges because values are influenced by magnetic field strength and sequence parameters [38].

T2 mapping assesses myocardial transverse relaxation time and serves as a marker of myocardial oedema. Increased T2 values have been reported in CA, particularly in AL amyloidosis, suggesting an inflammatory or injury-related component of myocardial involvement [45,46]. Elevated T2 values have also demonstrated prognostic significance in AL-CA and may assist in differentiating oedema from fibrosis in cases with elevated T1 values [46,47].

LGE remains a well-established technique for identifying myocardial involvement in CA. Amyloid fibril deposition results in marked expansion of the interstitial space, leading to increased gadolinium accumulation and delayed contrast washout compared with normal myocardium [48]. LGE, therefore, reflects both amyloid infiltration and fibrosis related to microvascular ischemia [49].

**Figure 2:**
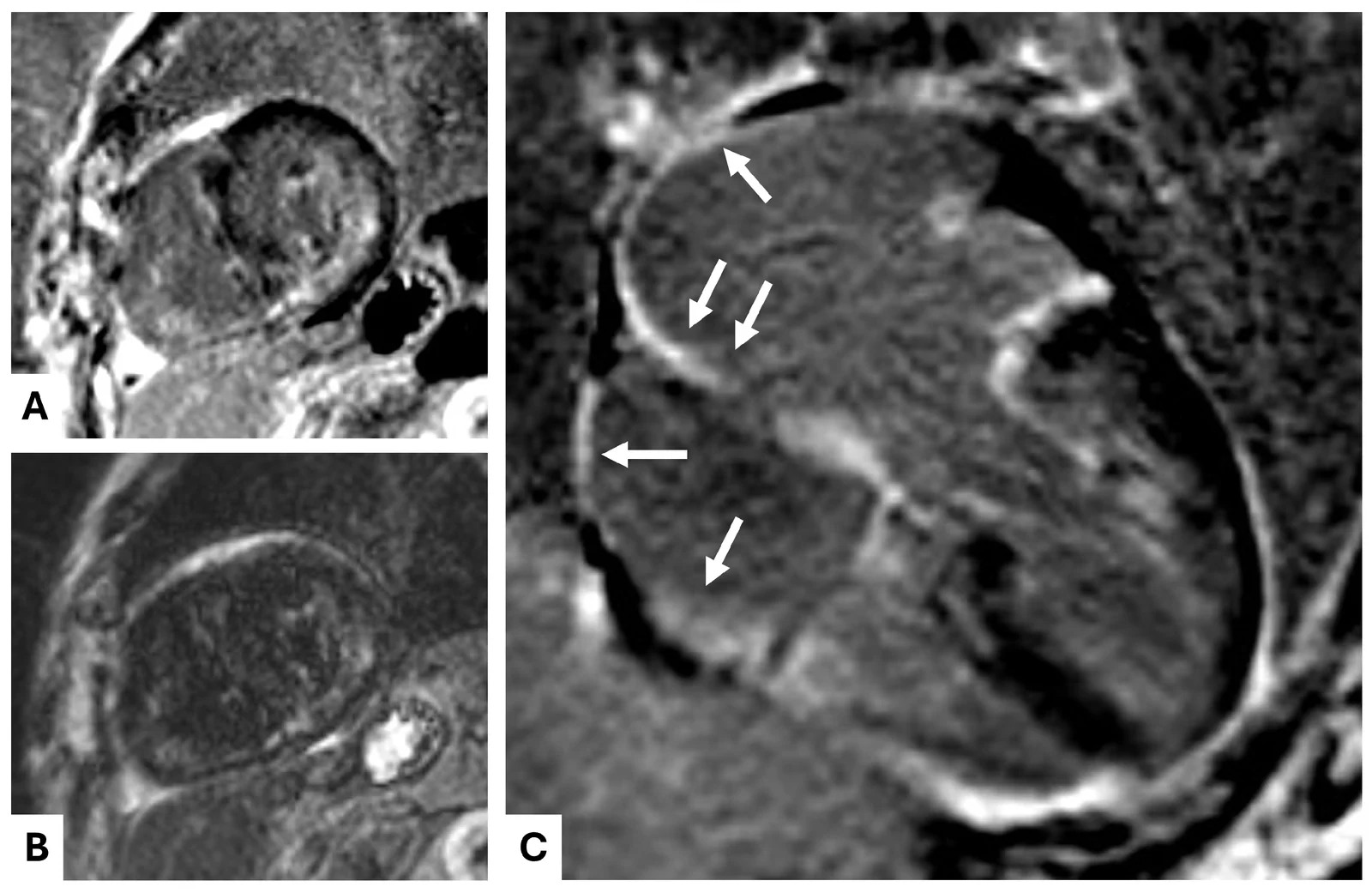
Representative cardiac magnetic resonance (CMR) findings in a patient with cardiac amyloidosis. (**A**) Short-axis basal view using phase-sensitive inversion recovery (PSIR)–late gadolinium enhancement (LGE), demonstrating non-ischemic transmural enhancement in the infero-lateral wall. (**B**) Corresponding magnitude image showing significant nulling failure of the remote myocardium; the superiority of the PSIR reconstruction (**A**) allows better signal discrimination and visualisation of the non-ischemic LGE pattern. (**C**) Four-chamber long-axis view on PSIR sequence revealing prominent bi-atrial LGE (arrows).

Historically, a diffuse global subendocardial enhancement pattern associated with abnormal myocardial and blood pool contrast kinetics was considered highly suggestive of CA (Figure 2A,B) [48]. The introduction of phase-sensitive inversion recovery (PSIR) techniques has improved reproducibility and allowed better delineation of myocardial involvement [50].

**Figure 3:**
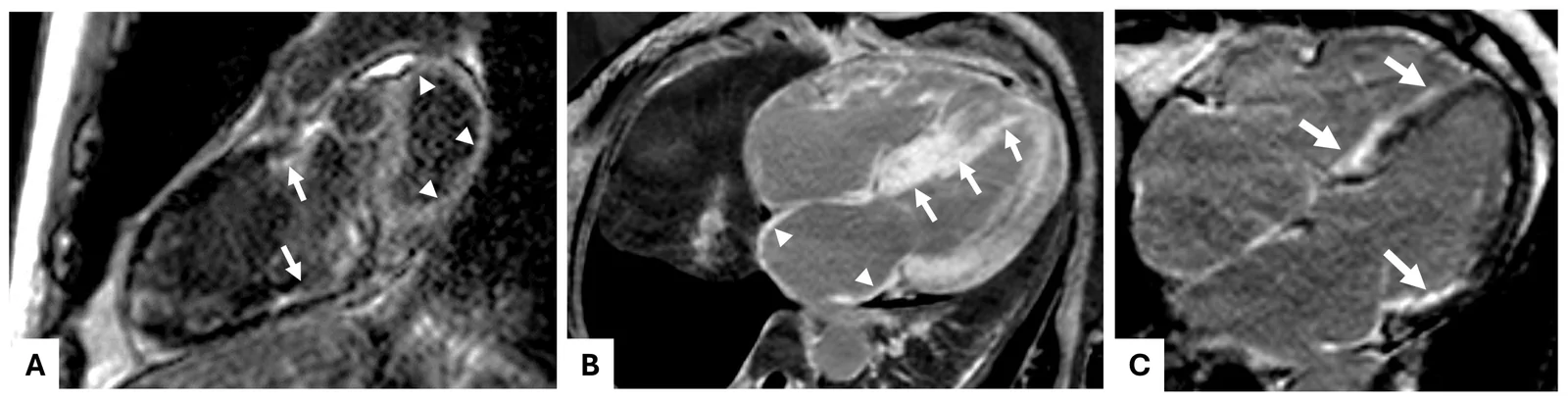
Cardiac magnetic resonance (CMR)–late gadolinium enhancement (LGE) patterns in cardiac amyloidosis. (**A**) Two-chamber view using phase-sensitive inversion recovery (PSIR) sequence showing non-ischemic subendocardial LGE confined to the basal segment of the left ventricle (arrows), accompanied by prominent left atrial LGE (arrowheads). (**B**) PSIR LGE image in another patient with cardiac amyloidosis displaying a characteristic apico-basal gradient, featuring transmural LGE in the mid-basal segments transitioning to subendocardial LGE in the apex (arrows) and difficulty in nulling the non-involved myocardium; extensive atrial LGE is also evident (arrowheads). (**C**) PSIR LGE image in another patient with cardiac amyloidosis displaying transmural LGE at basal interventricular septum and subendocardial LGE on the right ventricular side at mid-apical level.

Three characteristic LGE patterns are typically described: absence of enhancement, subendocardial enhancement, and transmural enhancement, reflecting a progressive continuum of amyloid infiltration [36]. A basal-to-apical gradient of LGE distribution has also been reported (Figure 3) [51].

Although differences in LGE distribution between AL and ATTR amyloidosis have been described, findings remain inconsistent, limiting its ability to reliably differentiate subtypes [37,52]. Overall, LGE demonstrates high diagnostic performance, with an estimated sensitivity and specificity of approximately 85% and 92%, respectively [53].

Importantly, LGE has strong prognostic implications. Transmural LGE is associated with the worst outcomes and identifies patients at high risk who may require closer monitoring and aggressive therapeutic strategies [36].

In addition to ventricular involvement, atrial LGE is frequently observed (Figure 2C) and correlates with atrial mechanical dysfunction, atrial fibrillation, and increased thromboembolic risk, representing an additional marker of disease severity and risk stratification [54]. Pericardial enhancement may also be present in advanced stages, reflecting amyloid infiltration [55].

Although concentric LV thickening with diffuse subendocardial LGE represents the classic presentation [36,48], atypical phenotypes are increasingly recognised and may cause diagnostic confusion. Wall thickening may be asymmetric—occasionally septal-predominant, mimicking hypertrophic cardiomyopathy—particularly in ATTR-CA [31] and may be mild or absent in early disease [35]. Likewise, LGE patterns may be atypical in the early stages of the disease. Initial amyloid infiltration may manifest as subtle, focal or patchy enhancement, or may be absent altogether, before the classic diffuse subendocardial and subsequently transmural patterns become established [36,48,56]. In this phase, parametric mapping is particularly informative, since native T1 and ECV are frequently already abnormal when LGE is still subtle or negative [35,48,49].

Several conditions producing increased LV wall thickness enter the differential diagnosis of CA. Hypertrophic cardiomyopathy typically shows asymmetric septal hypertrophy with patchy mid-wall LGE, often at the right ventricular insertion points, whereas native T1 and ECV are only mildly elevated and remain well below the values observed in CA [29,57]. Hypertensive heart disease produces concentric hypertrophy but is characterised by preserved or high ECG voltages relative to wall thickness and by only modest increases in native T1 and ECV [57]. Aortic stenosis may cause a comparable degree of hypertrophy, yet native T1 and ECV remain substantially lower than in CA [49,58]; notably, ATTR-CA frequently coexists with severe aortic stenosis in the elderly [16,58]. Anderson–Fabry disease is characterised by low native T1 values reflecting intracellular glycosphingolipid accumulation, together with basal inferolateral LGE [38]. Cardiac sarcoidosis produces patchy, often septal or subepicardial LGE, with frequent conduction disease and possible right ventricular involvement [8]. Key CMR discriminators favouring CA include markedly elevated ECV (typically >40%), high native T1, difficulty nulling the myocardium with abnormal gadolinium kinetics, and the apical-sparing strain pattern [14,35,38,48], whereas markedly reduced native T1 should specifically prompt consideration of Fabry disease [38]. CMR may also reveal extracardiac manifestations of systemic amyloidosis. These include bilateral pleural effusions, often disproportionate to the degree of ventricular dysfunction, hepatosplenomegaly with heterogeneous hepatic enhancement, renal cortical atrophy, and diffuse soft-tissue infiltration, such as macroglossia or salivary gland enlargement [56]. Bone marrow signal abnormalities suggestive of plasma cell dyscrasia may also be observed, particularly in AL amyloidosis [48].

**Figure 4:**
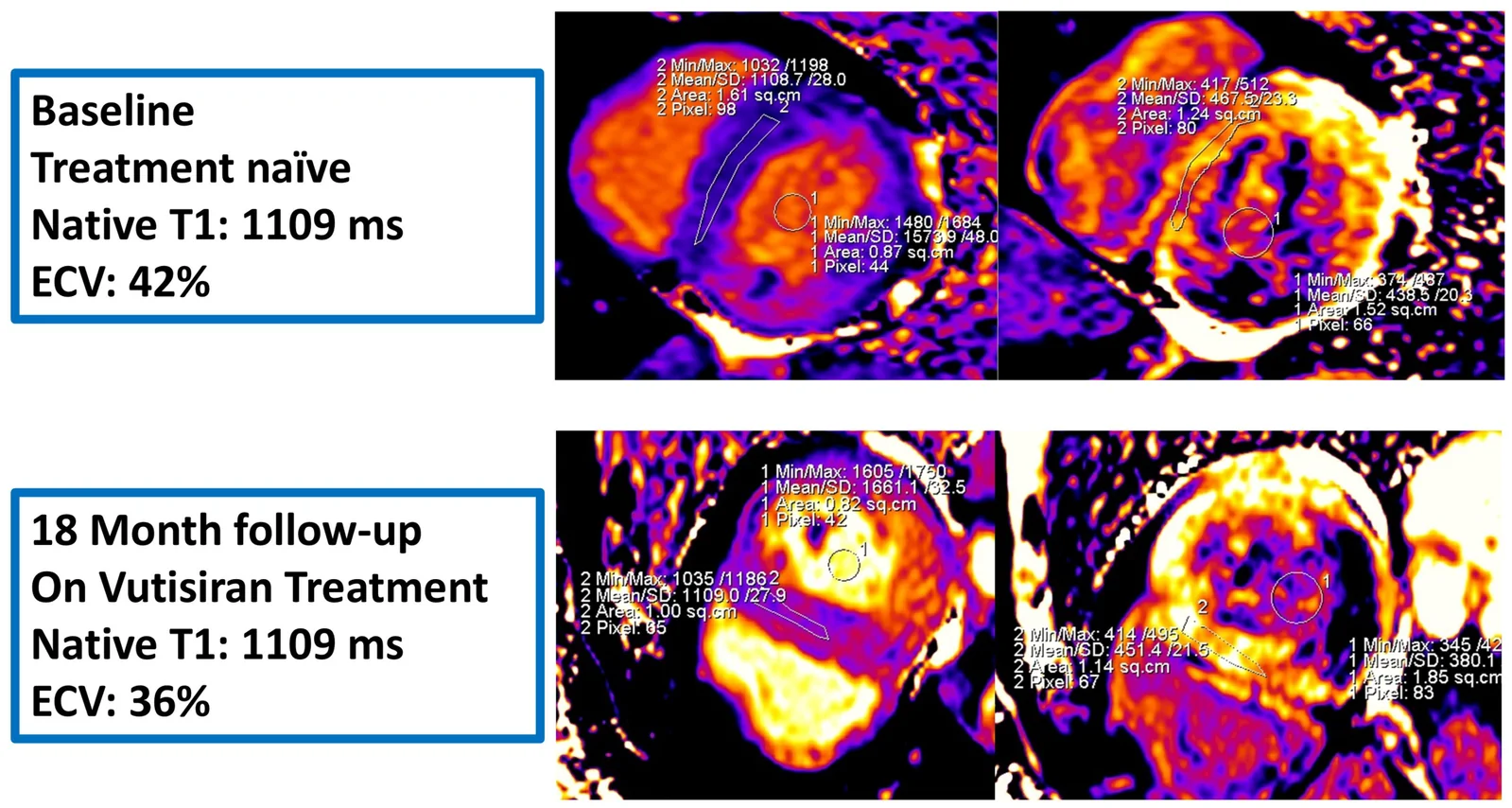
Cardiac magnetic resonance imaging of the same patient reported in Figure 2. Short-axis, mid-slice, at baseline without treatment (Naïve) (**top** panels, from left to right: native T1 and post-contrast T1 mapping) and 18-month follow-up after initiation of vutrisiran therapy (**bottom** panels, from left to right: native T1 and post-contrast T1 mapping) in a patient with TTR cardiac amyloidosis. At follow-up, a regression of ECV is observed.

Overall, CMR represents one of the most comprehensive imaging techniques for CA, providing simultaneous evaluation of morphology, function, tissue composition, and disease burden (Table 2). It plays a pivotal role in diagnosis, prognostic stratification, and increasingly in monitoring response to disease-modifying therapies [41] (Figure 4). Limitations include limited availability, higher costs, and contraindications to contrast administration in some patients. Currently, no uniform consensus exists regarding the optimal timing of serial CMR examinations, although most recommendations suggest follow-up imaging every 6–48 months or in the presence of clinical deterioration [59].

## 5. Computed Tomography

Myocardial ECV can be quantified by CT because iodinated contrast agents, like gadolinium chelates, distribute passively within the extracellular space, so that late myocardial iodine accumulation mirrors interstitial expansion [60]. Two technically distinct approaches are available. The first is applicable to conventional single-energy scanners and relies on a subtraction method: myocardial and blood pool attenuation are measured on a non-contrast acquisition and again on a late, equilibrium-phase acquisition, and ECV is derived from the change in attenuation corrected for the haematocrit. The second exploits spectral (dual-energy) acquisition, in which data collected at two different photon energy levels allow material decomposition and the direct generation of iodine density maps from a single late-phase scan, thereby obviating the need for a true non-contrast acquisition [60]. Spectral acquisition requires dedicated hardware (dual-source dual-tube systems, rapid kV switching, dual-layer detectors or, more recently, photon-counting detector CT) and is, therefore, less widely available than conventional CT [60,61]. Evidence for the single-energy approach is the most mature. Early pilot studies demonstrated increased myocardial attenuation on delayed contrast-enhanced CT images in patients with CA compared with controls [62]. Using equilibrium single-energy CT, subsequent investigations showed that CT-derived ECV is significantly higher in CA and correlates closely with ECV measured by CMR [63]. Prognostic relevance has since been established: in a large cohort of patients with systemic amyloidosis undergoing ECG-gated cardiac CT, CT-derived ECV was associated with myocardial remodelling and independently predicted mortality, supporting CT as a faster, cheaper and more widely available alternative to CMR, particularly in patients with cardiac devices or on dialysis [64].

Spectral (dual-energy) CT has been evaluated in parallel. Myocardial iodine concentration measured using dual-energy CT accurately distinguishes CA from other cardiomyopathies, with high diagnostic performance in small cohorts [65,66] A head-to-head comparison of the two techniques within the same patients with CA showed that global ECV derived from both single-energy and dual-energy acquisitions correlates closely with CMR-derived ECV, with good reliability for both and a numerically higher intraclass correlation for the dual-energy approach [5]. More recently, photon-counting detector CT has been shown to yield ECV values correlating excellently with CMR in patients with CA, with the single-energy technique showing no significant difference from CMR and the dual-energy technique only a small, clinically negligible underestimation, while allowing assessment of the coronary arteries within the same examination [61].

Although cardiac CT is not considered a first-line imaging modality for the diagnosis of CA, it may represent a valuable alternative when CMR is contraindicated or not feasible, such as in patients with severe claustrophobia or non-CMR-compatible implantable cardiac devices. In addition, CT-derived ECV assessment may be opportunistically obtained during clinically indicated scans. In particular, CT performed for pre-procedural planning of transcatheter aortic valve replacement (TAVR) has been proposed as a screening tool for CA in patients with severe aortic stenosis, a population in which the prevalence of concomitant amyloidosis has been reported to reach 10–15% [58].

Overall, while CT-based myocardial tissue characterisation remains investigational and limited by radiation exposure and iodinated contrast use, it holds promise as a complementary modality within a multimodality imaging framework, particularly in selected clinical scenarios where other imaging modalities are unavailable or impractical.

## 6. Other Non-Invasive Diagnostic Tools

Growing interest has recently focused on the development of non-invasive imaging techniques for the early detection of AL-related cardiac amyloidosis. Among these, positron emission tomography (PET) tracers originally developed for cerebral amyloid imaging have shown promising results.

Radiotracers such as ^18^F-florbetapir and ^18^F-florbetaben have demonstrated the ability to detect myocardial amyloid deposition. ^18^F-florbetapir shows myocardial uptake in both ATTR-CA and AL-CA and may allow quantitative assessment of myocardial amyloid burden, suggesting a potential role in disease monitoring and evaluation of treatment response [19,67].

More recently, ^18^F-florbetaben has demonstrated differential binding properties between amyloid subtypes. Preliminary data suggest a higher binding affinity for AL amyloid fibrils, resulting in more persistent myocardial tracer retention and slower washout compared with ATTR-CA or other phenocopies. These findings suggest a potential role for ^18^F-florbetaben in the non-invasive identification of AL-CA, although further validation in larger prospective cohorts is required [68].

Despite these encouraging results, PET imaging in CA remains investigational, and its clinical application is currently limited to research settings.

Table 3 compares the practical strengths, limitations, current indications and resource requirements of each modality.

## 7. Clinical Implications

Early identification of cardiac amyloidosis is crucial in both AL and ATTR subtypes, as cardiac involvement is the main determinant of prognosis [69]. The introduction of disease-modifying therapies has substantially changed the management of CA, with several treatments demonstrating significant survival benefit. Importantly, therapeutic efficacy is greatest when treatment is initiated in the early stages of cardiac involvement [7,70].

Risk stratification in CA currently relies primarily on circulating biomarkers [71]. In AL-CA, established staging systems incorporate N-terminal pro-B-type natriuretic peptide (NT-proBNP), cardiac troponins, and serum free light chains [72]. In ATTR-CA, prognostic stratification is mainly based on NT-proBNP levels and the estimated glomerular filtration rate (eGFR) [73].

Beyond biomarkers, multimodality cardiac imaging provides incremental prognostic information. Echocardiographic parameters such as global longitudinal strain, atrial strain, and right ventricular function have been consistently associated with clinical outcomes. Similarly, CMR-derived markers, including late gadolinium enhancement patterns, ECV quantification, and myocardial mapping techniques, offer robust prognostic value and allow comprehensive assessment of myocardial disease burden. In particular, ECV has emerged as a promising quantitative biomarker not only for diagnosis and risk stratification, but also for longitudinal disease monitoring. Recent data suggest that myocardial amyloid burden measured by CMR tracks the continuum of ATTR-CM and may help identify disease progression over time, with ECV providing a dynamic estimate of cardiac amyloid load rather than a purely static descriptor of phenotype [74]. In addition, reductions in CMR-derived ECV after patisiran therapy have provided evidence of cardiac amyloid regression, supporting the concept that CMR can detect treatment-related changes in myocardial amyloid burden [41]. Likewise, serial CMR studies in tafamidis-treated ATTR-CM suggest that ECV may help monitor therapeutic response and refine prognosis, with a change in ECV emerging as an independent predictor of adverse outcomes in treated patients [75,76,77]. Table 4 summarises the role of multimodality imaging in CA across the clinical continuum.

The integration of imaging findings with clinical and laboratory parameters is, therefore, essential not only to refine diagnosis and risk stratification, but also to guide therapeutic decision making and monitor disease progression and response to treatment in the era of disease-modifying therapies.

## 8. Future Perspectives

The role of imaging in CA is likely to shift further from description towards quantification and decision making. The demonstration that ECV regresses under disease-modifying therapy [40,41] and that it independently predicts outcome and refines the interpretation of haematological response [1] raises the prospect of quantitative imaging serving as a surrogate endpoint in clinical trials; this will first require standardised acquisition and post-processing, scanner-specific reference ranges [38], agreement on the minimal clinically important change in ECV, and prospective confirmation that treatment-induced changes translate into better outcomes.

Emerging techniques may extend both reach and access: amyloid-binding PET tracers allow direct quantification of myocardial amyloid burden and could identify AL involvement, for which no equivalent to bone scintigraphy currently exists [67,68], while photon-counting detector CT combines spectral tissue characterisation, coronary assessment and lower radiation exposure within a single examination [61]. Both await wider availability, standardised quantification and outcome data.

Artificial intelligence, meanwhile, may address the persistent problem of underdiagnosis. Models applied to the standard 12-lead ECG detect CA with good discrimination and can identify affected patients years before clinical diagnosis [78], and fully automated pipelines achieve comparable performance using either ECG or echocardiographic input [79]. Prospective validation in real-world populations and attention to generalisability across scanners and centres remain essential.

## 9. Conclusions

Multimodality imaging is central to the contemporary management of cardiac amyloidosis across the entire disease continuum. Echocardiography remains the gatekeeper for raising suspicion; bone scintigraphy enables non-invasive diagnosis of ATTR-CA once monoclonal gammopathy is excluded; and CMR provides unmatched tissue characterisation for early detection, risk stratification and, increasingly, quantitative monitoring of treatment response through ECV. Emerging modalities—dual-energy (spectral) CT and amyloid-binding PET tracers—may broaden diagnostic reach and quantitative monitoring but require further validation. The principal value of imaging lies in its integration with clinical and biochemical data, enabling earlier diagnosis, more precise prognostication, and objective assessment of therapeutic efficacy in the era of disease-modifying therapies.

## Figures and Tables

**Table 1:** Echocardiographic features/red flags of cardiac amyloidosis.

Echocardiographic Feature/Parameter	Typical Findings in CA	Clinical Significance
LV morphology	Increased LV wall thickness (usually concentric; occasionally asymmetric) with increased LV mass index	Reflects interstitial amyloid infiltration rather than true myocyte hypertrophy
	Small or normal LV cavity size	Consistent with restrictive physiology
LV systolic function	Preserved or mildly reduced LVEF in early stages	LVEF may remain normal despite advanced infiltration
	Reduced stroke volume	Early marker of systolic dysfunction; associated with worse prognosis
LV diastolic dysfunction	Restrictive filling pattern (E/A > 2)	Hallmark of increased myocardial stiffness
	Markedly elevated E/e′	Suggests elevated filling pressures; prognostic marker
Myocardial strain	Reduced GLS with relative apical sparing (“cherry-on-top”)	Sensitive marker of early dysfunction; highly suggestive of CA; associated with worse prognosis
Right ventricular involvement	RV wall thickening	Indicates advanced/systemic infiltration
	Reduced TAPSE or RV strain	Independent adverse prognostic marker
Atrial involvement	Bi-atrial enlargement	Reflects chronic elevation in filling pressures and direct atrial infiltration
	Reduced LA reservoir strain	Marker of atrial mechanical dysfunction and thromboembolic risk
	Atrial thrombus despite sinus rhythm	Suggests amyloid-related atrial myopathy
Valvular and IAS involvement	Thickened AV valves and interatrial septum	Supportive infiltrative feature
	
Pericardial findings	Mild-to-moderate pericardial effusion	Common supportive finding; reflects serosal involvement
Associated findings raising suspicion	Low-flow, low-gradient AS with preserved EF	Strongly associated with ATTR-CA in elderly patients
	LV hypertrophy with low ECG voltages	Classic red flag for infiltrative cardiomyopathy

**Table 2:** CMR features of cardiac amyloidosis.

CMR Feature/Parameter	Typical Findings in CA	Clinical Significance
LV morphology	Concentric (occasionally asymmetric) LV hypertrophy with small ventricular cavity	Imaging phenotype of restrictive cardiomyopathy
LGE pattern	Diffuse global subendocardial enhancement	Highly suggestive of CA; confirms myocardial infiltration
	Transmurality of LGE	Marker of advanced disease; strong predictor of mortality
Gadolinium kinetics	Difficulty nulling myocardium; myocardium brighter than blood pool	Indicates high amyloid burden and abnormal contrast kinetics
Native T1 mapping	Markedly elevated native T1 values	Quantitative marker of amyloid infiltration; useful when contrast contraindicated
Extracellular volume (ECV)	Markedly expanded (often >40–50%)	Best CMR correlate of amyloid burden; strong diagnostic and prognostic value; useful for therapy monitoring
T2 mapping	Elevated T2 values (often higher in AL)	Reflects myocardial oedema/inflammation; associated with worse prognosis
Right ventricular (RV) involvement	RV free wall thickening and LGE	Common in advanced disease; adverse prognostic marker
Atrial involvement	Bi-atrial enlargement and atrial LGE	Associated with high risk of atrial fibrillation, atrial dysfunction, and thromboembolic risk
Feature tracking (strain)	Reduced GLS with apical sparing	Detects early dysfunction despite preserved EF
Extra-cardiac findings	Pleural effusion	Common supportive finding; reflects systemic involvement
	Ascites	Suggests advanced systemic disease
	Hepatosplenomegaly	More common in AL; indicates extracardiac infiltration
	Abnormal liver/spleen gadolinium kinetics	Suggests systemic amyloid deposition

**Table 3:** Practical comparison of imaging modalities in cardiac amyloidosis.

Modality	Main Strengths	Main Limitations	Current Indication and Optimal Timing	Diagnostic/Prognostic Value	Availability and Cost
Echocardiography	Bedside; no radiation or contrast; assesses haemodynamics; apical-sparing strain highly suggestive	Operator-dependent; cannot distinguish AL from ATTR; insensitive to subclinical infiltration	First-line; entry point of the pathway, in any unexplained LV thickening or HFpEF	Diagnostic: suggestive, not confirmatory Prognostic: GLS, LA and RV function predict mortality	Universal; low
Bone scintigraphy (^99m^Tc-DPD/PYP/HMDP + SPECT)	Non-invasive confirmation of ATTR-CA without biopsy; high specificity once gammopathy is excluded	Does not detect AL; mandatory concurrent exclusion of monoclonal gammopathy; uptake grade not prognostic; not suited to monitoring	Confirmatory step once CA is suspected: Perugini grade ≥ 2 with negative gammopathy screen establishes ATTR-CA	Diagnostic: high for ATTR Prognostic: limited	Wide (nuclear medicine); low–moderate
CMR	Best tissue characterisation; LGE, native T1, ECV, T2 and strain in one examination; quantitative and reproducible; detects early infiltration	Limited availability; gadolinium in severe renal failure; devices and claustrophobia; scanner-specific reference ranges	Second-line after echocardiography when CA is suspected or the diagnosis is uncertain; differential diagnosis; baseline quantification before therapy and serial ECV for monitoring	Diagnostic: high (LGE, native T1, and ECV) Prognostic: strong and independent (ECV and transmural LGE)	Moderate; high
Cardiac CT (single-energy or spectral)	Fast; feasible with devices or dialysis; opportunistic on TAVR-planning scans; coronary assessment in the same study	Radiation; iodinated contrast; no LGE equivalent; limited outcome data; spectral/photon-counting hardware not widely available	Alternative when CMR is contraindicated or unavailable; opportunistic screening on pre-TAVR CT	Diagnostic: good (ECV vs. CMR) Prognostic: emerging	Conventional: wide, low; spectral/PCCT: limited, high
Amyloid PET (^18^F-florbetapir/^18^F-florbetaben)	Binds to amyloid directly; detects both AL and ATTR; potential for quantitative monitoring	Investigational; limited tracer availability; no standardised quantification or thresholds; scarce outcome data	Research setting; potential future role in AL-CA and in treatment monitoring	Diagnostic: promising Prognostic: limited data	Very limited; high

**Table 4:** Role of multimodality imaging in cardiac amyloidosis across the clinical continuum.

Modality/Parameter	Early Diagnosis	Risk Stratification	Treatment Monitoring
Echo—LV wall thickness, low-voltage/wall-thickness discordance, valvular and IAS thickening	Raises suspicion (red flags); insensitive to subclinical disease	↑ LV mass; ↓ stroke volume; low-flow state linked to outcome	Limited (morphology changes slowly)
Echo—GLS with apical sparing (“cherry-on-top”)	Sensitive; may precede EF decline; highly suggestive of CA	Reduced GLS predicts mortality	Emerging; may track response
Echo—LA strain and RV function (TAPSE; RV strain)	Adjunctive; early atrial/RV dysfunction	Strong independent predictors of mortality	Limited data
Bone scintigraphy (^99m^Tc-DPD/PYP/HMDP + SPECT)	Non-invasive ATTR diagnosis	Uptake grade not prognostic	Not established
CMR—LGE	High accuracy; subendocardial → transmural	Transmural LGE = worst prognosis	Qualitative; limited for serial use
CMR—native T1	Elevated early	Correlates with severity; prognostic	Promising surrogate endpoint
CMR—ECV	Best quantitative correlate of amyloid burden; detects early infiltration	Strong independent predictor	Established emerging role; ECV regression with disease-modifying therapy
Dual-energy (spectral) CT—CT-ECV	Alternative when CMR contraindicated; opportunistic (TAVR-planning CT)	Limited data	Investigational
Amyloid PET (^18^F-florbetapir/^18^F-florbetaben)	Detects AL and ATTR	Limited data	Potential quantitative monitoring

## Data Availability

No new data were created or analysed in this study.

## Supplementary Materials

The following supporting information can be downloaded at https://www.mdpi.com/article/10.3390/jcdd13080401/s1: Video S1: Echocardiography clip. 4-chamber view.
